# Flexible multilevel nonvolatile biocompatible memristor with high durability

**DOI:** 10.1186/s12951-023-02117-5

**Published:** 2023-10-13

**Authors:** Xiaoping Chen, Xu Zhao, Xiaozhong Huang, Xiu-Zhi Tang, Ziqi Sun, Da-Long Ni, Hailong Hu, Jianling Yue

**Affiliations:** 1 Powder Metallurgy Research Institute, Central South University, Changsha, 410083 China; 2 School of Chemistry and Physics, QUT Centre for Materials Science, Queensland University of Technology, Brisbane, QLD 4001 Australia; 3 Department of Orthopaedics, Shanghai Key Laboratory for Prevention and Treatment of Bone and Joint Diseases, Shanghai Institute of Traumatology and Orthopaedics, Ruijin Hospital, Shanghai Jiao Tong University School of Medicine, Shanghai, 200025 China; 4 Research Institute of Aerospace Technology, Central South University, Changsha, 410083 China; 5 State Key Laboratory of Powder Metallurgy, Hunan Key Laboratory of Advanced fibers and Composites, State Key Laboratory of High Performance Ceramics and Superfine Microstructure, Research Institute of Aerospace Technology, Central South University, Changsha, 410083 China

**Keywords:** Implantable memristors, AlOOH nanosheets, Information storage, Multilevel

## Abstract

Current protein or glucose based biomemristors have low resistance-switching performance and require complex structural designs, significantly hindering the development of implantable memristor devices. It is imperative to discover novel candidate materials for biomemristor with high durability and excellent biosafety for implantable health monitoring. Herein, we initially demonstrate the resistance switching characteristics of a nonvolatile memristor in a configuration of Pt/AlOOH/ITO consisting of biocompatible AlOOH nanosheets sandwiched between a Indium Tin Oxides (ITO) electrode and a platinum (Pt) counter-electrode. The hydrothermally synthesized AlOOH nanosheets have excellent biocompatibility as confirmed through the Cell Counting Kit-8 (CCK-8) tests. Four discrete resistance levels are achieved in this assembled device in responsible to different compliance currents (I_CC_) for the set process, where the emerging multilevel states show high durability over 10^3^ cycles, outperforming the protein-based biomemristors under similar conditions. The excellent performance of the Pt/AlOOH/ITO memristor is attributed to the significant role of hydrogen proton with pipe effect, as confirmed by both experimental results and density functional theory (DFT) analyses. The present results indicate the nonvolatile memristors with great potential as the next generation implantable multilevel resistive memories for long-term human health monitoring.

**Graphical Abstract:**

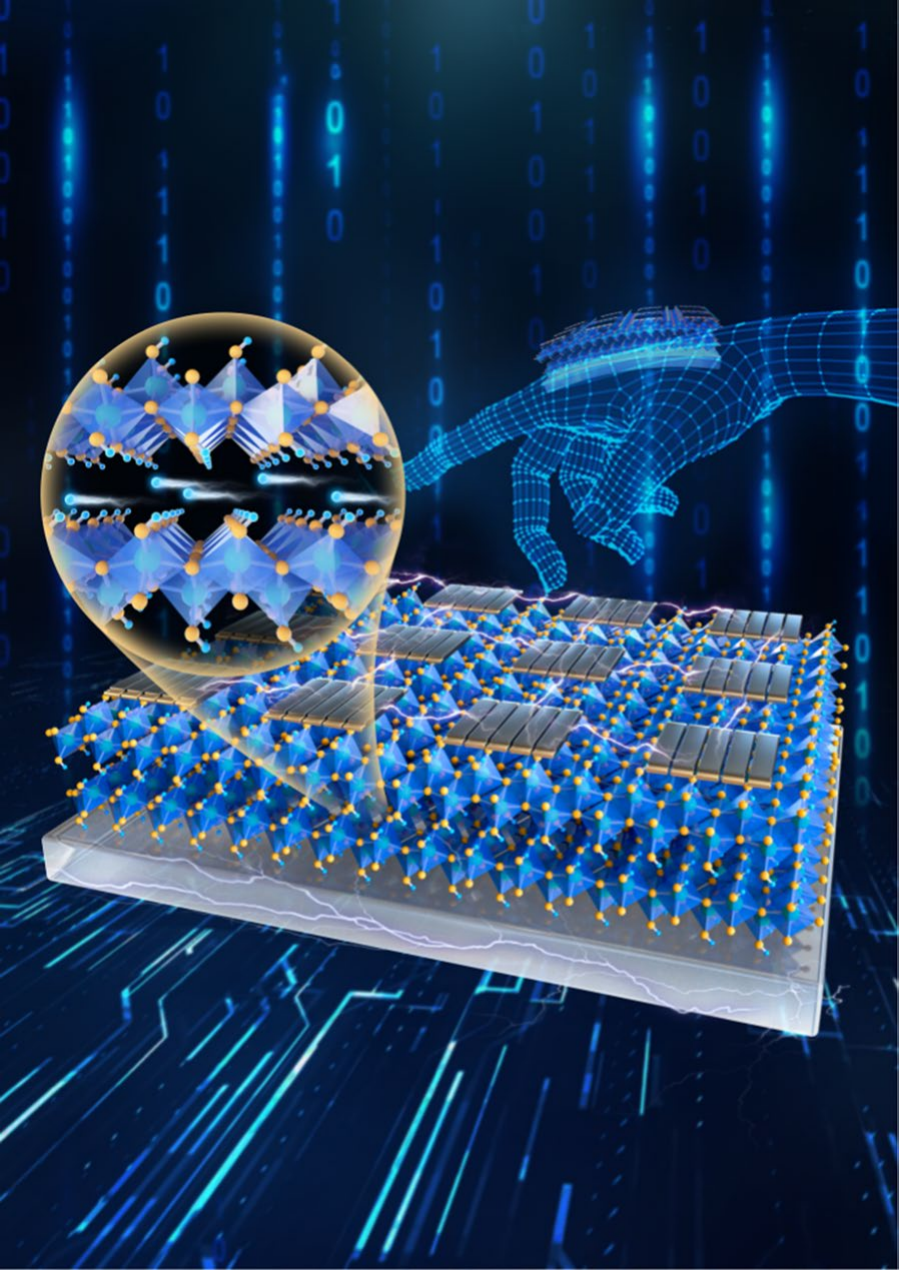

**Supplementary Information:**

The online version contains supplementary material available at 10.1186/s12951-023-02117-5.

## Introduction

Memristors are one of the most promising candidates for the next-generation of non-volatile memories in logic operations and brain neural function modeling [1–3]. The increasing demand for the real-time and long-term monitoring of human health in recent years has stimulated great interest in implantable memristors with feature resistance-switching characteristics and biocompatibility [4, 5]. Presently, a wide range of materials have been demonstrated to show resistance switching properties, including inorganic metal-oxides, metal chalcogenides, and organic natural biomaterials. Specifically, natural biomaterials as proteins or glucose as representatives have been regarded as the leading candidates to build the next generation of implant memristors, owing to their excellent biocompatibility [6]. Most current protein or glucose based biomemristors (Additional file 1: Fig. S1), [7–17] however, exhibit poor resistance switching performances, such as low endurance (< 500 cycles), high variability, and usually need to match active electrodes (Ag, Al, W, Cu), which have substantially hindered the development of implantable memristor devices. Meanwhile, the solubility characteristic of proteins or glucose renders this class of biomemristors, which are unsuitable for long-term information storage in the living body. In addition, the existing biomemristors generally demonstrate only single-level or mostly not exceed three-level resistive switching characteristics, which limit the data storage density. It is acknowledged that the multi-level cell characteristics of memristors responded towards the I_CC_ during set operation can offer a unique opportunity to achieve more than 2-bits of storage in a single device [18, 19], and thus significantly enhancing the data storage density [20]. Therefore, to meet the rigorous demands of future implantable memristors, it is imperative to develop novel biomemristors with highly sensitive and durable resistive switching performance and good biocompatibility.

In this work, a flexible, highly sensitive and durable biomemristor based on a layered structure material, AlOOH nanosheets, is innovated to realize multi-resistive memristive characteristics and good biocompatibility. Compared with other biomemristors, AlOOH-based memristors present highly responsible multilevel resistance states and environmental stability, and exhibit a great potential for next generation long-term storage and information classification of human body for implantable health monitoring. The key component, Boehmite or aluminium oxide hydroxide (γ-AlOOH), possesses a large number of out-of-plane hydrogen atoms to form hydrogen bonding networks and maintain the integrity of the layered structure [21, 22] and allows rapid proton transport through the hydrogen-bond proton-transfer channels [23]. Owing to its particular structure, γ-AlOOH has shown versatile potential applications in the fields of biomedicine, environmental remediations, electrochemical energy conversions, optical nanodevices, etc. It is noted that γ-AlOOH has an excellent biosafety record, which is a common inorganic adjuvant among various human vaccines approved by the American Food and Drug Administration (FDA) [24, 25]. Meanwhile, γ-AlOOH is also an advanced antibacterial material [26]. All these features indicate that γ-AlOOH could be a very promising candidate material for implantable devices. However, there few reports on the application of γ-AlOOH into the implantable memristors to date, and the mechanisms in regards to proton transfer and the contributions of the proton conduction with hydrogen to the resistance switching effect remain unclear. Recently, Yao et al. demonstrated a resistive random-access memory (RRAM) device based on a metal–organic framework (MOF) material, FJU-23-H_2_O [23]. Ascribed to a voltage-gated proton conduction with hydrogen bond pathways instead of oxygen-ion migration, resistive switching properties have been verified, which may bring some clues for the understanding of γ-AlOOH-based memristors. Therefore, contributed by the migration of hydrogen protons in shaping the resistance switching behaviors, a flexible multilevel nonvolatile biocompatible γ-AlOOH-based memristor is designed for highly sensitive, bio-safe, and environmentally durable implantable health monitoring. It is expected that this study will inevitably offer new insights into the development of next generation flexible implantable biomemoritors for durable and biosafe healthy monitoring.

## Results and discussion

**Fig. 1:**
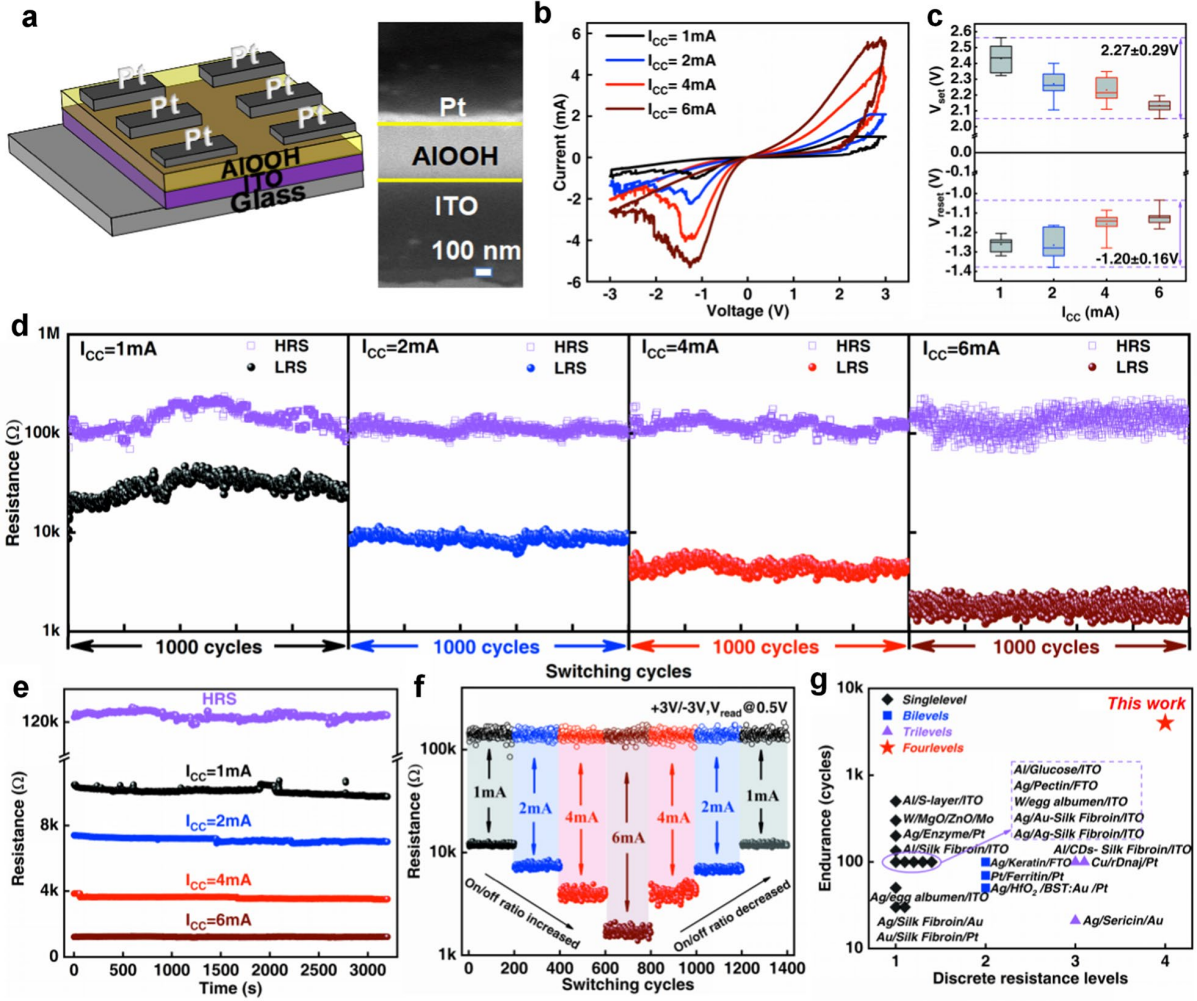
Memristive behavior of Pt/AlOOH/ITO device. **a** Two-terminal memristive device in Pt/AlOOH/ITO configuration (left) and cross-sectional SEM image of the Pt/AlOOH/ITO device (right). **b** Four different I-V curves of the device at different levels of Icc (1 mA, 2 mA, 4 mA and 6 mA), and each I-V curves has a different LRS/HRS ratio. **c** Switch voltage distributing in its 4 states, a total of 120 cycles. **d** Repeatability test of the HRS and LRS values of the fabricated device under four different orders of magnitude of I_CC_ (1 mA, 2 mA, 4 mA and 6 mA). The endurance of the device over 10^3^ cycles at “On” and “Off” states respectively in all four states. **e** The retention time of the four resistance states all exceeded 3.2 × 10^3^ s. A read voltage of 0.5 V was used for the measurement. **f** The resistance of the device over 1400 cycles under the Icc sweep of 1 mA → 2 mA → 4 mA → 6 mA → 4 mA → 2 mA → 1 mA. **g** The comparison of the endurance and discrete resistance levels between AlOOH and other representative RRAM implantable materials, as presented in Additional file 1: Table S1

Schematic of memristive device structure and cross-sectional SEM image of the Pt/AlOOH/ITO device are presented in Fig. 1a. The memristor comprises fluorine-doped tin oxide (ITO) as one electrode and platinum (Pt) (≈85 nm) as the other electrode with AlOOH film (≈300 nm) sandwiched in between. The response characteristics of the Pt/AlOOH/ITO memristor were recorded under different compliance currents and applied bias voltages. As shown in Fig. 1b, four different I-V curves of the configurated device of Pt/AlOOH/ITO can be distinctly achieved to reach the compliance currents (I_CC_) of 1 mA, 2 mA, 4 mA, and 6 mA, respectively under the DC voltage sweep of 0 V → 3 V → 0 V → −3 V → 0 V. The four different I-V curves in Fig. 1b show that a lower reset current can be obtained by using a lower I_CC_. This proves that the use of different I_CC_ in the set process for the AlOOH based memristor contributes to multilevel low resistance states, showing its applicability to multi-bit storage in one device. At the same time, the device has extremely high sensitivity to I_CC_ changes. And the resistance value of the device changes significantly only by changing a small I_CC_ change. In addition, the device exhibits the same multilevel low resistance states (Additional file 1: Fig. S2a–c) under the DC voltage sweep of 0 V → 4 V → 0 V → − 4 V → 0 V. This confirms the feasibility that the storage density of the device can be further improved.

The switching between low and high resistance states is found to be highly stable. Figure 1c shows the switching voltage distributions in its four states over a total of 120 cycles. It is found that V_set_ is concentrated at 2.27 ± 0.29 V and V_reset_ is concentrated between − 1.20 ± 0.16 V under the DC voltage sweep of 0 V → 3 V → 0 V → -3 V → 0 V. Therefore, it can be concluded that the switching voltage of the device in the four states is relatively confined within a narrow value range, demonstrating the desired stability of the device.

To further evaluate the reliability of the four states of the device, its endurance and retention tests were carried out under four different currents (I_CC_ = 1 mA, 2 mA, 4 mA and 6 mA), as shown in Fig. 1d. Note that the test read voltage is 0.5 V and the loading voltage are 3 V and − 3 V respectively. Figure 1d shows that the resistance of high resistance state (HRS) shows no pronounced variation with different I_CC_, whereas the resistance of low resistance state (LRS) significantly decreases with the increasing I_CC_, which further confirms the device has a high sensitivity for the change of I_CC_. As a result, the endurance of the device under each individual I_CC_ of 1 mA, 2 mA, 4 mA and 6 mA has surpassed 1000 cycles, with no degradation being observed, showing the strong robustness and reliability of the four resistance states in this operating device. Figure 1e shows the retention characteristic of the corresponding resistance state of the device under four different orders of I_CC_ (1 mA, 2 mA, 4 mA and 6 mA), where the resistance was read every 1 s with a voltage of 0.5 V. It is found that the retention time of the four resistance states all exceeded 3200 s, indicating its multiple stable intermediate resistance states. Therefore, RRAM device with AlOOH shows great potential in multi-density storage as evidenced by both retention and endurance performance test.

To evaluate the effectiveness of the device in multi-resistance state storage, the cyclability of the device under different I_CC_ was also studied. The results show that the device achieves four discrete resistance levels that can be apparently distinguished by using different I_CC_ for the set process and further investigation shows that four resistance levels of AlOOH based memristor can be switched stably under each individually applied I_CC_ of 1 mA, 2 mA, 4 mA and 6 mA, as shown in Fig. 1f. Surprisingly, the device can stably circulate 1400 cycles under the Icc sweep of 1 mA → 2 mA → 4 mA → 6 mA → 4 mA → 2 mA → 1 mA, which is distinctly higher than the values (about 21–22 cycles) previously reported on protein and related similar types of biomemristors [8, 14]. This verifies that four storage states of the device are completely reversible. To this end, the potential of AlOOH based resistance switching memory device for multi-level memory has been demonstrated.

To show the potential applications of our fabricated AlOOH based device, the devices based on AlOOH and some representative implantable or bio-compatible materials including the inorganic and organic media are compared in Fig. 1g and Additional file 1: Table S1 [7–17, 27–34]. First, AlOOH demonstrates the highest endurance value in implantable memristors, which also shows its superiority compared to that of other reported organic and in-organic based devices. Secondly, the AlOOH based device can successfully function and survive up to four levels of I_CC_, followed by multiple stable intermediate resistance states, which can be repeated with high endurance (> 10^3^ cycles). Other implantable or bio-compatible materials can only survive up to max three levels of I_CC_ and do not show multi-resistance states that can be switched stably. The set voltage of AlOOH device is about 2.27 V without the conventionally applied electroforming voltage, which is lower than most biological materials. Among the comparable candidates, Pt/AlOOH/ITO device thus demonstrates excellent endurance performance with the highest cycling values (four states all > 10^3^), as well as multilevel memory operation and good retention performance under the small I_CC_ range. In addition, the device shows a competitive transparency of over 88% under visible light. Therefore, AlOOH-based memristors show great utility in wearable or implantable applications owing to their stable memristive performance.

**Fig. 2:**
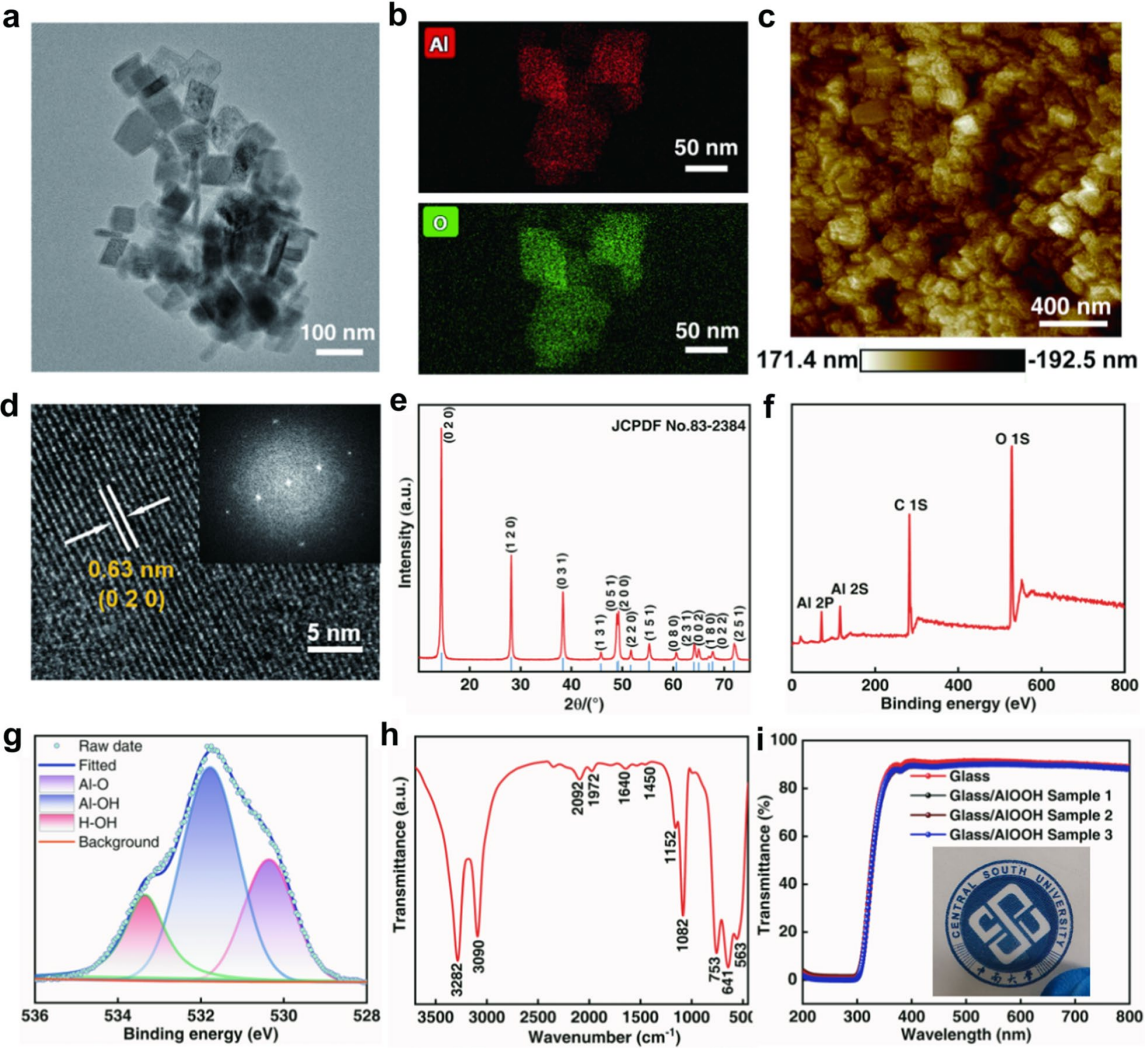
Structure characterization of AlOOH. **a** Low-magnification TEM image of AlOOH nanosheets. **b** The corresponding element mapping of Al and O in AlOOH nanosheets. **c** AFM topography image of AlOOH film illustrating distribution and arrangement of AlOOH nanosheets in the film. **d** High-resolution TEM image showing the crystal lattice of AlOOH. The AlOOH crystal surface spacing is 0.63 nm, corresponding to the (0 2 0) crystal orientation. Inset: the corresponding FFT pattern. **e** XRD patterns of AlOOH film. **f** XPS analysis of AlOOH film. **g** O 1s core level spectra for the AlOOH film. **h** FTIR images of AlOOH film. **i** Transmittance spectra of Glass/AlOOH in the visible region; the CSU logo beneath the devices is clearly visible without any distortion owing to the excellent transparent properties of the device structure

Figure 2a shows the morphology of AlOOH nanosheets under low-magnification TEM, whose monolithic morphology is close to a square shape (Additional file 1: Fig. S3a). Additional file 1: Fig. S3b shows the particle size of AlOOH nanosheets falling in the range of 75–85 nm in statistics based on analysis of Fig. 2a. Figure 2b shows the corresponding element mapping of Al and O in AlOOH nanosheets whose element ratio is approximately 1:2. Additional file 1: Fig. S3c is the image of HAADF-STEM, where AlOOH nanosheets are stacked with a layered structure. Specifically, from Additional file 1: Fig. S3d–f and Fig. 2c, the surface of AlOOH film prepared through the drip coating method is relatively flat, where AlOOH nanosheets in the film layer do not indicate the obvious agglomeration, showing a stacking uniformly. Figure 2d depicts the high-resolution TEM image of the AlOOH crystal lattice of AlOOH. The AlOOH crystal surface spacing is 0.63 nm, which is corresponding to the (0 2 0) plane of AlOOH. The corresponding fast Fourier transform (FFT) pattern indicates that the basal plane of the AlOOH is the (0 2 0) plane (inset of Fig. 2d). These are consistent with the X-ray diffraction (XRD) pattern result presented in Fig. 2e. The XRD result suggests that observed diffraction peaks are all consistent with standard JCPDF No. 83-2384, indicating the pure AlOOH phase. The obtained AlOOH belongs to the orthorhombic cell with lattice parameters of a = 3.69 Å, b = 12.21 Å, and c = 2.87 Å [35]. Narrow reflection peaks of (020), (120) and (031) are demonstrated, which are corresponding to the formation of the γ-AlOOH phase with a high crystallinity. Therefore, high purity of AlOOH is achieved with uniform structure.

The chemical state of atoms in the prepared sample is studied by X-ray photoelectron spectroscopy (XPS). The XPS spectra of Al 2p, Al 2s, C 1s and O 1s regions in AlOOH is shown in Fig. 2f. Carbon decomposition occurs during the charging correction, as can be revealed by the C 1s XPS spectrum. Two peaks at 74.5 and 119 eV are Al 2p and Al 2s in AlOOH [36]. The peaks at 531.7 and 532.8 eV are attributed to the lattice oxygen (O 1 s) of AlOOH and the oxygen of surface hydroxyl (OH) groups, respectively [37]. The O 1s spectra is deconvoluted into three peaks, which are corresponding to the three types of oxygen species. In Fig. 2g, the peaks at 530.6–530.7, 532.0–532.1, and 533.1–533.2 eV are attributed to bulk oxygen from the crystal structure (Al-O, 28.4%), surface hydroxyl groups (Al–OH, 53.1%), and adsorbed water on the surface (H-OH, 18.5%) [38]. High amount of hydroxyl groups on AlOOH is reflected by the O_OH_/O_Latice_ ratio of 65%. In conjunction with the crystal structure of AlOOH, AlOOH is confirmed with a large number of hydrogen bonds. At the same time, AlOOH contains almost no oxygen vacancies from the O 1 s spectra deconvoluted, which is different from traditional oxide materials.

To verify the rationality of the peak splitting of O 1s, Fourier transform infrared (FTIR) is used to further characterize the prepared AlOOH nanosheets, as shown in Fig. 2h. The bands at approximately 1082 cm^−1^ and 1152 cm^−1^ represent the (OH)-Al=O asymmetric stretching and the O–H bending, respectively, which are the typical characteristic of AlOOH. The absorption peaks at 563 cm^−1^, 641 cm^−1^ and 753 cm^−1^ are attributed to the stretching vibration mode of AlO_6_ [39]. The weak band at 1640 cm^−1^ is attributed to the stretching and bending modes of adsorbed water molecules despite the very weak absorbance in the AlOOH nanostructure spectrum, indicating that the number of physically adsorbed water molecules is quite few [40]. The asymmetric and symmetric stretching of the interlayer OH groups are observed at 3282 and 3090 cm^−1^, respectively [41]. This is consistent with the result of the O 1 s spectra deconvoluted of XPS. Interestingly, the transmittance curve of the AlOOH film demonstrates a transparency of over 88% under visible light at a wavelength range of 350–800 nm (in Fig. 2i). AlOOH film is competitive in transmittance when compared to that of other transparent materials, such as ZnO, TaO_x_, protein, etc. [15, 17, 42, 43].

**Fig. 3:**
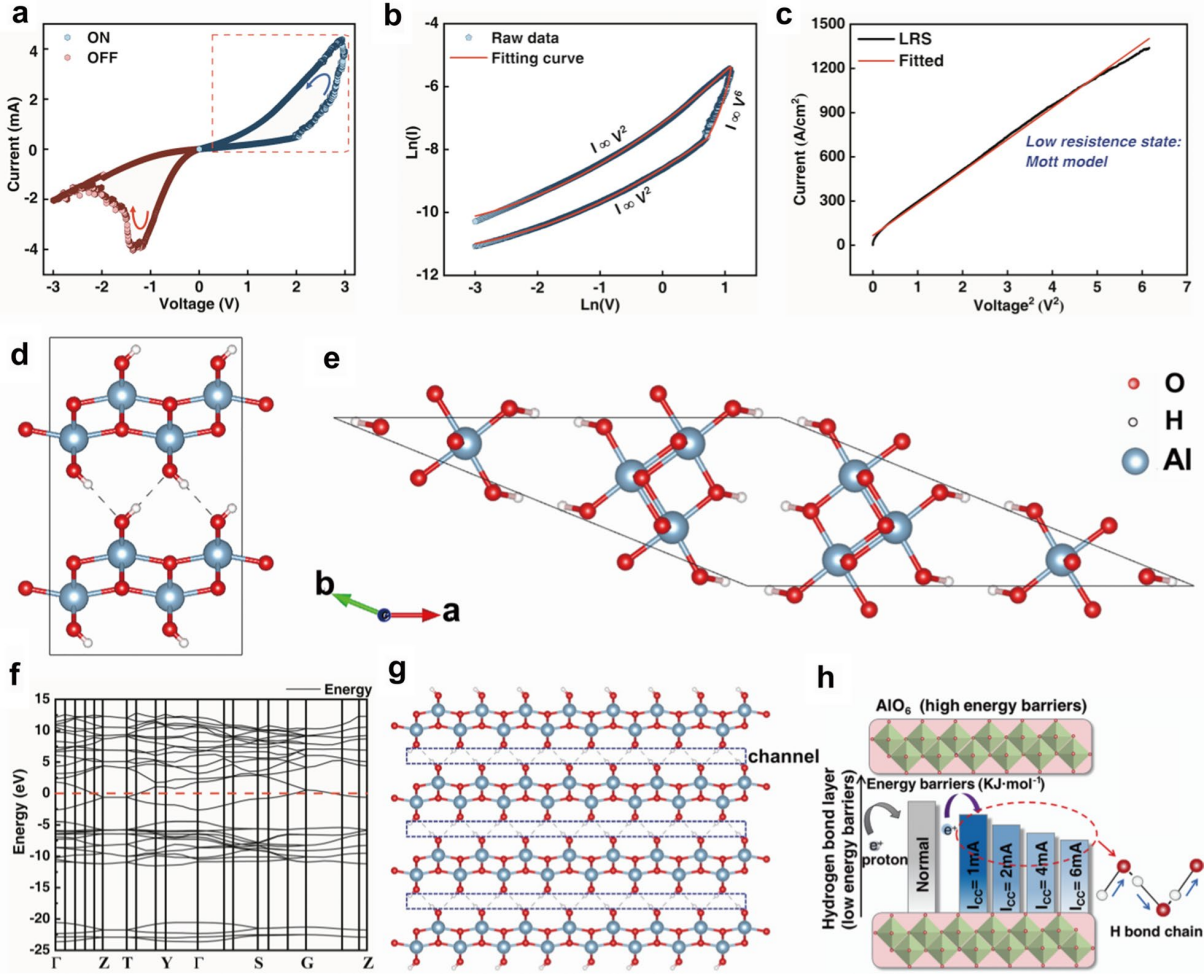
Switching mechanism of Pt/AlOOH/ITO/PDMS device. **a** Typical current versus voltage (I–V) curves of the Pt/AlOOH/ITO/PDMS device in voltage sweeping mode at room temperature. A compliance current of 4 mA is used in set process. **b** The fitting slopes of the I-V curve for the red square area in **a**. **c** Fitting curve at the LRS portion showing Mott − Gurney behavior as the conduction mechanism. **d**, **e** The unoptimized (**d**) and fully optimized (**e**) models of AlOOH configuration respectively. **f** The band structure of AlOOH structure. **g** The formation of the pipe effect in the AlOOH material. **h** Schematic diagrams illustrating the proposed memristive switching mechanisms in AlOOH memristors. The reduction of O–H bond length with increasing I_CC_ could enhance the proton migration by reducing the potential barrier, thereby raising the conductivity

Figure 3a shows the cyclic current–voltage (I-V) characteristics of the device, which was obtained at the 4 mA by the DC voltage sweep of 0 V → 3 V → 0 V → -3 V → 0 V. The device was initially in the OFF state, exhibiting high resistance. When the applied voltage exceeded the set voltage (V_set_ ~ 2 V), the device state switched from high resistance state to low resistance state. Subsequently, the low resistance state was maintained until the applied voltage exceeded the reset voltage (V_reset_ ~ − 1.2 V), and the device gradually changed to the high resistance state. Finally, the device completely returned to the high resistance state at the applied voltage ~ − 2.5 V. In general, the threshold voltage of the device is relatively small. Besides, it is worth noting that the device does not requires a positive-forming process with a large voltage sweep. This is correlated with the resistance change mechanism, because it is different from the conventional memristors which rely on oxygen vacancy conduction [44].

To reveal the physical process inside the device, the I-V curves is plotted in dual-logarithmic scales, where the fitting result of the I-V curve agrees with the space charge limited current (SCLC) model in this device (Fig. 3b), followed by the subsequent voltage scan curves of the device under different I_CC_ which share the same model. To reveal the intrinsic resistive switching behavior, the carrier mobility was calculated in the low resistance state. The transport behavior was fitted based on the Mott–Gurney law (Fig. 3c) [45].

1 $$\mathrm{I}=\frac{9}{8}{{\upvarepsilon }_{r}\upvarepsilon }_{0}\upmu \frac{{V}^{2}}{{d}^{3}}$$

where *μ* is the carrier mobility, *ε*_*r*_ is the relative permittivity, *ε*_*o*_ is the permittivity of free space, and *d* is the distance between electrodes. According to the fitting curve, the carrier mobility was determined to be ≈240 cm^2^ V^−1^ s^−1^, by using *ε*_*r*_ = 8.5 and *d* = 0.3 µm. This value is comparable to the carrier mobility reported in semiconductor oxides from Additional file 1: Table S2 [46, 47].

Moreover, the mobility of between AlOOH layers is quantified through acoustic phonon scattering mechanism, where the structural optimizations and electronic structure calculations are performed based on density functional theory (DFT) as implemented in the Vienna Ab Initio Simulation Package (VASP) code based on the projector augmented wave (PAW) method with a cutoff energy of 600 eV^2^. The Brillouin zone sampling is carried out using the (3 × 3 × 1) Monkhorst–Pack grids for surface and Gamma for the structure. The adopted crucial parameters are concluded as follows: convergence tolerance of energy is 1 × 10^–5^ eV, maximum force 0.002 eV·Å^−1^, and maximum displacement 0.002 Å. The rest of the calculation details are described in the experimental part. The pristine and fully optimized models of AlOOH configuration are shown in Fig. 3d and e, respectively. The carrier mobility is calculated as follows (The detailed calculation can be referred to formula unit conversion in the Additional file):

2 $$\mu =\frac{e{\hslash }^{3}{\rho S}_{l}^{2}}{{k}_{B}T{m}^{*}{m}_{d}{E}_{l}^{2}}$$

where *S*_*l*_ is the area of xy plane for the supercell, *E*_*l*_ is deformation potential constant, *m*_*e*_ is effective mass of electron, and *m*_*d*_ is average effective mass. The carrier mobility of AlOOH is determined to be 247 cm^2^ V^−1^·s^−1^ according to the band structure of AlOOH structure in Fig. 3f, by using *e* = 1.6 × 10^–19^ C, ℏ = 1.05 × 10^–34^ J·S, *K*_*B*_ = 1.38 × 10^–23^ J K^−1^, *pS*_*l*_^*2*^ = 4.6 × 10^8^ J m^−2^, m^*^ = 0.19 kg, *m*_*d*_ = 0.516 kg, *E*_*l*_ = 1.01 × 10^–19^ J, which is consistent with the results of the carrier mobility calculated in the low resistance state, confirming the significance of hydrogen bond being responsible for the memristive behavior. The high carrier mobility is attributed to the formed pipe effect in the multilayer AlOOH material (Fig. 3g), which improves the transport properties of hydrogen proton. Moreover, the band structure where the K point defined in reciprocal lattice of the primitive cell is folded into point sitting at the Γ–Y–S branch of the supercell also confirms the pipe effect phenomenon. The result shows that the material with a very narrow band gap behaves the same as the band structure of conductor to some extent.

To further reveal and confirm the effect of hydrogen bonding on the memristive performance, AlOOH nanosheets was calcined at 600 °C for 2 h to avoid the effect of the hydrogen bond and consequently obtained Al_2_O_3_ nanosheets with the same shape. The XRD pattern result and morphology of Al_2_O_3_ nanosheets were presented in Additional file 1: Fig. S5a and b. Through the electrical measurements, it is found that the memristive performance of the Al_2_O_3_ device is completely different from that of the AlOOH device after eliminating the existence of hydrogen bonding, showing none multi-resistive state (Additional file 1: Fig. S5c), which further confirms the effect of hydrogen bonding on the memristive behavior through the experimental aspect.

Furthermore, the hydrogen ion in the hydrogen bond chain is highly itinerant with complex structural features, which can be feasible for proton conduction applications. Energy barriers of hydrogen transfer path on both perfect and vacancy-containing crystal structures were computed through the theoretical aspect, whose energy barriers were generally below 21 kJ mol^−1^ in a perfect crystal, and 14 kJ mol^−1^ in a hydrogen vacancy-containing structure [48]. These low energy barriers are the desired indicators of high proton conductivity of AlOOH even at room temperature.

From the information gathered so far, we propose the working principles for the resistive switching in AlOOH memristor. Initially, under the action of an electric field, the hydrogen protons migrate, and part of the hydrogen vacancies are occupied by the injected hydrogen protons. As the applied voltage increases, the traps are gradually filled in the order of energy levels. Then, the barrier height gradually decreases, which leads to a decrease in impedance. As the impedance decreases, the channel current increases. Finally, the high and low resistance states are transformed. In the reverse sweep, the hydrogen protons trapped in the deep energy levels cannot be thoroughly released with decreasing applied voltage, which indicates that a portion of the traps are always filled and that the diminished barrier height cannot be recovered to the initial states, leading to the emergence of non-volatile behavior. The specific energy band diagram of the device is shown in Additional file 1: Fig. S6. The main mechanism causing proton transport is the Grotthuss mechanism [49], which is the proton transporting to a nearby site through hydrogen bonds. On the other hand, a secondary proton transport mode is ascribed to the protons and adsorb water molecules forming hydronium ions for transporting [50]. The existence of less adsorbed water has been proved by the XPS and FTIR characterization in Fig. 2.

The mechanism of multi-level switching by regulating I_CC_ is generally explained by the proposed formation of conductive filaments or conductive paths composed of oxygen vacancies or metal ions [14, 51]. As the increase of applied I_CC_, thicker conductive filaments or more conductive paths are created inside devices, which further decrease the resistance value of the low-resistance state, consequently showing the multi-resistance state. According to the performance under multi-configurated Icc as well as the aforementioned resistance switching mechanism of the AlOOH device, it is obviously not feasible to explain the phenomenon of multi-level switching through the conventional filament mechanism. Therefore, another applicable and confirmed mechanism to explain the multi-level switching behavior for this device has been reasonably proposed. The conductivity difference of multilevel low resistance states is attributed to the change of the O–H bond length between AlOOH layers under different I_CC_. When the device operated under high compliance current, the O–H bond length will decrease, resulting in the weaker bonding of hydrogen atoms and enhancing the proton migration by lowering the energy barriers [52], thus increasing the conductivity (Fig. 3h). This is also applicable to explain the phenomenon that the device has a more stable multi-level switching than that of the traditional conductive filament memristor, showing the cyclic use of multi-resistance states.

**Fig. 4:**
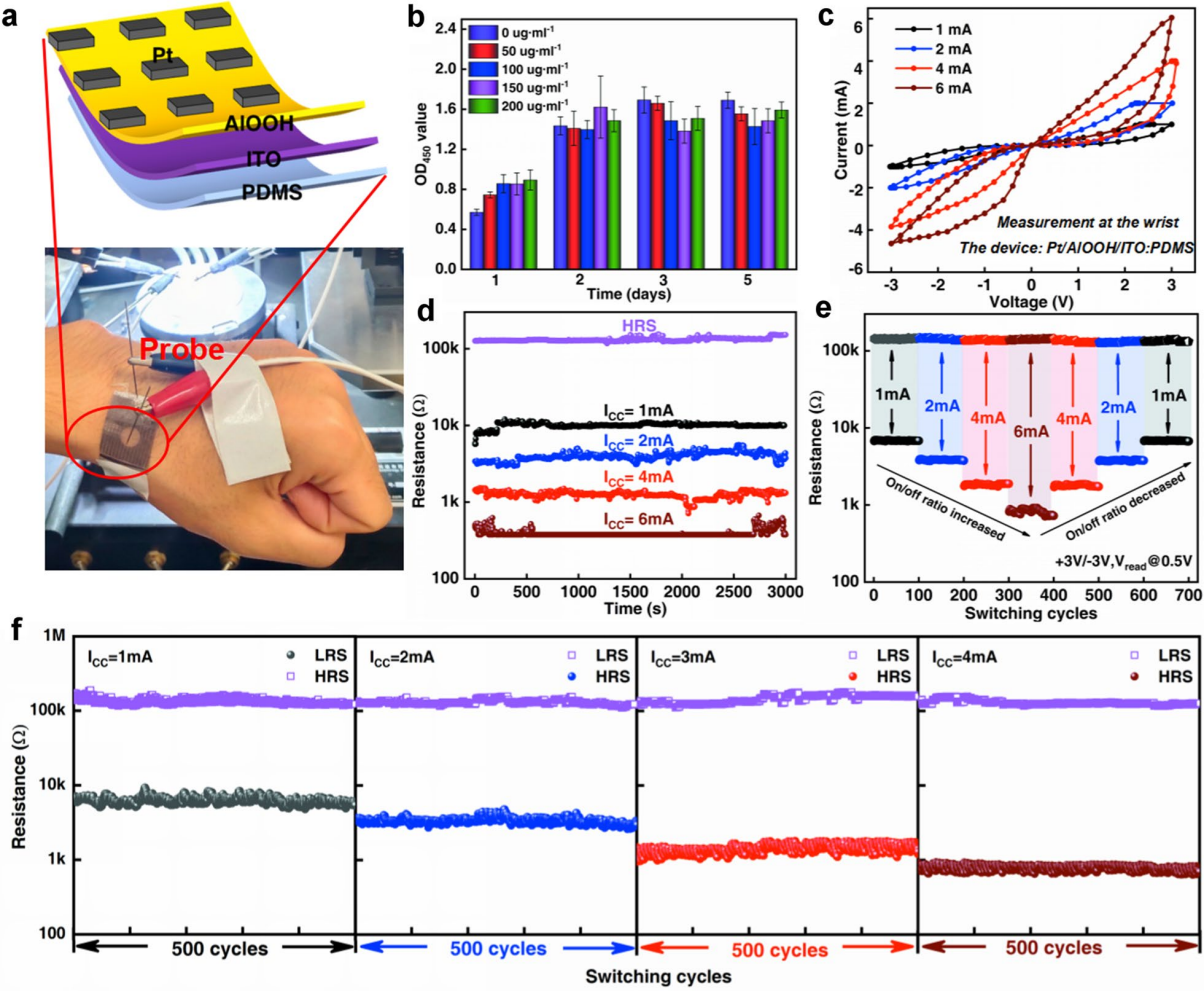
Flexible Pt/AlOOH/ITO:PDMS memristive device characterization. **a** Schematic illustration of AlOOH flexible memristive device and the physical display of the flexible device. **b** The CCK-8 results for AlOOH nanosheets on different samples for 5 days. **c** Four different I-V curves of the device at different levels of Icc (1 mA, 2 mA, 4 mA and 6 mA). **d** The retention time of the four resistance states all exceeding 3 × 10^3^ s. A read voltage of 0.5 V was used for the measurement. **e** Pt/AlOOH/ITO:PDMS memristive device stably circulate 700 cycles and the four storage states are completely reversible. **f** Repeatability test of the HRS and LRS values of the fabricated device under four different orders of magnitude of I_CC_ (1 mA, 2 mA, 4 mA and 6 mA)

Flexible resistance switching memory (RRAM) device with good biocompatibility is highly desirable due to their potential application in wearable and implantable electronics. These flexible AlOOH devices are insoluble compared with conventional biomemristors, so it is suitable for long-term detection and storage of human healthy. By evaluating the high performance of the AlOOH nanosheets prepared through hydrothermal method, RRAM devices with multilevel Nonvolatile states is achieved. As a feasibility test, the ITO glass substrate was replaced by ITO-coated polydimethylsiloxane (PDMS) substrate with good flexibility to fabricate flexible memristor, while other remaining device fabrication processes and characterization studies were the same as described earlier, as illustrated in Fig. 4a.

In addition, to confirm the excellent biocompatibility of AlOOH nanosheets, the CCK-8 test is performed with detailed analysis as shown in Fig. 4b and Additional file 1: Table S3. The optical density (OD450) value measured at a wavelength of 450 nm showed the proliferation activity of the experimental group and the control group gradually increased with the increase of the incubation time, which confirms the non-toxic nature of AlOOH nanosheets for Raw264.7 cells. In addition, when the concentration of the AlOOH nanosheets solution was 200 ug·mL^−1^, the cell activity still did not change significantly and demonstrated no obvious dose effect, showing good biocompatibility. Therefore, the desired biocompatibility of AlOOH is showing the feasibility as a promising candidate material for wearable and implantable memristors.

Even in the case of flexible devices, four different memory states can be clearly distinguished with nonvolatile character, as shown in Fig. 4c. In addition, the flexible devices show the strong robustness and reliability of the four resistance states in this operating device. Figure 4d shows the retention time of the four resistance states with values exceeding 3 × 10^3^ s. And the device can stably circulate 700 cycles under the Icc sweep of 1 mA → 2 mA → 4 mA → 6 mA → 4 mA → 2 mA → 1 mA (Fig. 4e). Moreover, the endurance of the device under each individual I_CC_ of 1, 2, 4 and 6 mA has surpassed 500 cycles, with no degradation being observed (Fig. 4f). The four resistance states of the device can perfectly reflect the health of the human body. Taking the level of human hormone secretion as an example, the four states of the memristor can perfectly correspond to the five states of hormone secretion: trace amount (undetectable, I_CC_ < 1 mA), low (I_CC_ = 1 mA), normal (I_CC_ = 2 mA), high (I_CC_ = 4 mA), and excessive (I_CC_ = 6 mA). The flexible biomemristor Pt/AlOOH/ITO/PDMS with other representative flexible biomemristors is compared in Additional file 1: Fig. S7 with respect to some crucial performance. Specifically, this flexible biomemristor Pt/AlOOH/ITO/PDMS has significant advantages in terms of cycle persistence and multi-bits of storage. Therefore, the device can simultaneously complete the information storage and the classification due to the multi-resistance states. In the future, the monitoring sensor composed of the memristors is expected to be realized, which can independently complete the integration of monitoring, storing, and classifying at the same time. It is expected to complete the human health monitoring and substantially reduce the incidence of major diseases.

The previous CCK-8 experiment results (Additional file 1: Table S3) showed that AlOOH showed excellent biocompatibility. In order to verify the feasibility of the application of the AlOOH flexible device in human body, the AlOOH flexible device was implanted in mice for biological experiments. The AlOOH flexible device was implanted subcutaneously in mice for long-term evaluation of its biological safety.

**Fig. 5:**
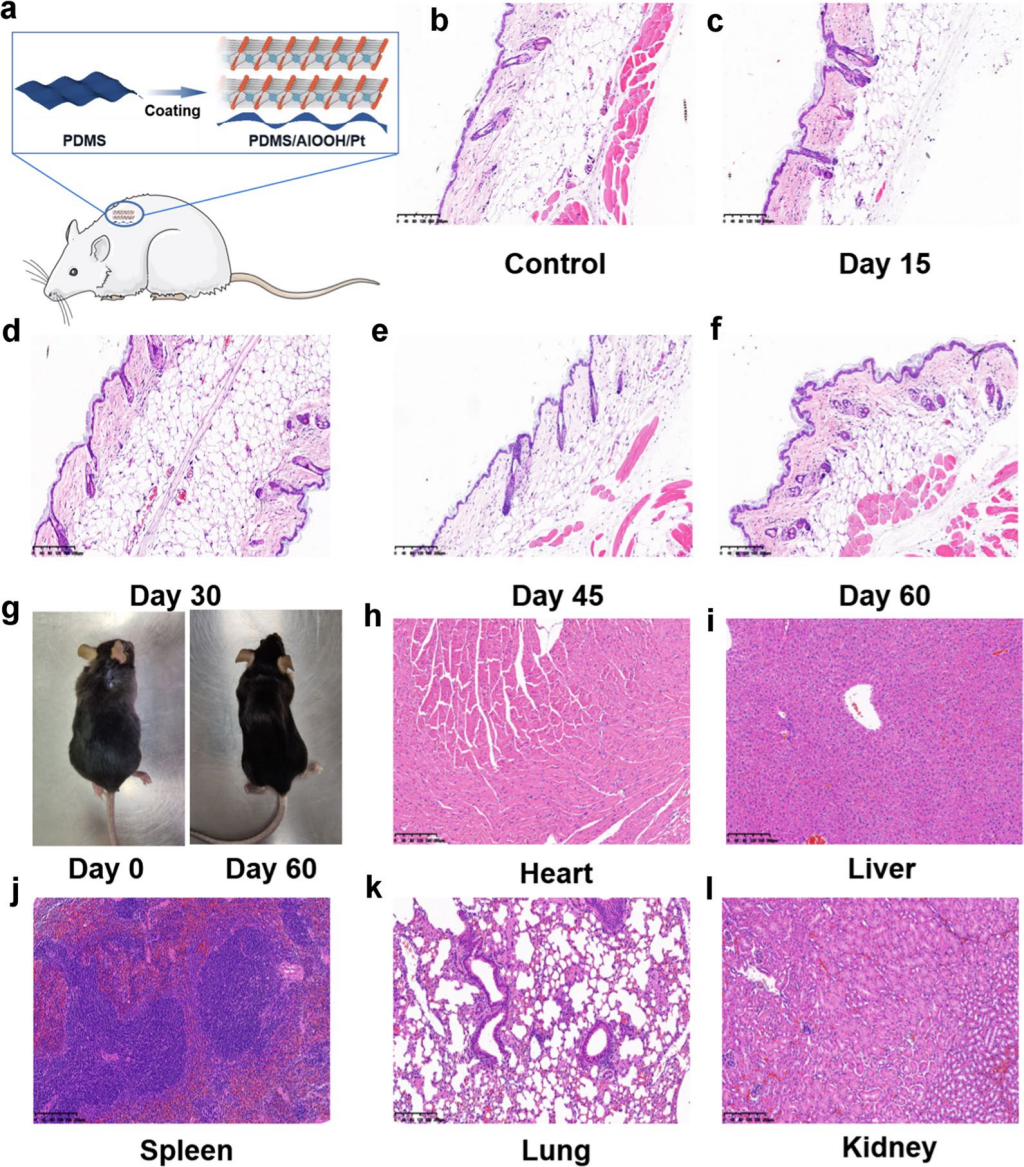
Histological analysis of all major organs of mice after 60 days of implantation of AlOOH flexible device and skin tissue analysis of implanted sites at various stages after implantation of AlOOH flexible device: **a** schematic diagram of implanted AlOOH flexible device in mice; **b**–**f** mouse skin tissues in contact with AlOOH flexible devices collected after 15, 30, 45 and 60 days; **g** mice after implantation of AlOOH flexible devices at 0 and 60 days; **h**, **i** histological analysis of heart, liver, spleen, lung and kidney after 60 days of implantation of AlOOH flexible device

For the biocompatibility study, five male C57BL/6 mice aged 6 weeks were used. For subcutaneous implantation, make a small incision at the back of the neck and insert the AlOOH flexible device into the wound. Then, suture the incision with absorbable suture, as shown in Fig. 5a. The skin tissue in contact with the AlOOH flexible device was collected from mice after incubation for 15, 30, 45 and 60 days to evaluate the biocompatibility of the AlOOH flexible device. The negative control is the skin tissue collected from mice that have never undergone any surgery or other experiments. All skin tissues were further stained with hematoxylin and eosin (H&E) for histological analysis, as shown in Fig. 5b–f. The experimental results showed that no obvious immune cell infiltration was observed in the surrounding tissues on the 15th, 30th, 45th and 60th days after implantation, indicating that the AlOOH flexible device has good compatibility with the host. In addition, the skin tissue near the AlOOH flexible device did not show dense fibrous capsule formation after 60 days, similar to the negative control skin tissue. This proves that AlOOH flexible memristor has potential for human application.

Finally, in order to evaluate whether the AlOOH flexible device is harmful to the main organs, samples of spleen, liver, lung, rein and heart were collected after 60 days of implantation and fixed with polyformaldehyde (in 4% PBS) for histological analysis. All the main organs of the heart, liver, spleen, lung and kidney of the mice after 60 days of implantation of the AlOOH flexible device were collected for H&E staining, as shown in Fig. 5g–i. No injury or inflammatory reaction was found in all these major organs. Although the test time in this part is only 2 months in mice, and there is no detailed characterization of the biological safety performance of the blood and other aspects of mice, it is the first of its kind to carry out the biological safety test in mice from the device level. The experimental results preliminarily confirmed that AlOOH flexible memristor has good biological safety and has the potential of implantable application in human body.

At present, research on biomemristors is mostly limited to the search for memristor materials. Biomaterials that can manufacture biomemristors mainly include natural biomaterials represented by proteins and inorganic materials with good biocompatibility. Natural biomaterials represented by proteins have become an important research direction in memristors due to low cost and degradability. However, there are many factors that affect the memristor characteristics of biomemristors represented by proteins during the preparation process, resulting in unstable memristors [7–9]. In addition, the working principles for the resistive switching in similar biomemristors are controversial and difficult to unify [14, 15]. The inorganic materials used in the preparation of biomemristors are mainly composed of oxides, and the switching mechanism is mainly related to various defects such as oxygen vacancies [46]. The number and type of defects directly affect the characteristics of biomemristors, but it is difficult to stably control the distribution and quantity of defects during the production process. The switching mechanism of AlOOH biomemristor is relatively clear, and there are few defects that affect the memristor characteristics, making the memristor characteristics stable. Therefore, AlOOH is a excellent biomemristor material. However, in order to meet the biocompatibility requirements of real-time and long-term monitoring of human health, it is necessary to further set up control experiments for comprehensive and detailed characterization, including the analysis of whether there is edema in cells from high magnification histology and the need for blood tests, statistics of whether various data such as hormones and biological enzymes are within the normal range, and finally carry out clinical experiments.

In conclusion, AlOOH nanosheets prepared by hydrothermal method initially show the stable resistance switching performance for nonvolatile memory. For the configurated Pt/AlOOH/ITO devices, performance of multi-level resistance switching memory has been achieved under different I_CC_ during the set process, with four states showing both high endurance (> 10^3^ cycles) and reliable retention (> 3.2 × 10^3^ s). The memristive behavior of the device can be attributed to the hydrogen proton transport. Moreover, the flexible Pt/AlOOH/ITO:PDMS memristors is designed to achieve a high-performance memory device with excellent biocompatibility. These outstanding characteristics show that our flexible AlOOH memristive device is a promising candidate for applications in inexpensive flexible bio-compatible electronics. This work provides a new direction for the material selection of wearable and implantable memristors, where metal hydroxide represented by AlOOH is expected to be the potential candidate for building the next generation of wearable and implantable memristors.

## Experimental section

### Material synthesis [53, 54]

The 1 mol L^−1^ NaOH solution was slowly dropped into a 250 mL three-necked flask containing 0.025 mol L^−1^ AlCl_3_ solution. The solution was adjusted to pH = 10, and the ultrasonic treatment was performed while mechanically stirring for 30 min. After the reaction was completed, the suspension was put into a reaction kettle lined with polytetrafluoroethylene, the reaction temperature was adjusted to 180 ℃, and the temperature was kept for 24 h. After the reaction was completed, it was washed and centrifuged three times with ethanol and deionized water, and then centrifuged at 60 ℃. After drying in 60 ℃, AlOOH nanosheets were finally obtained.

### Device fabrications

AlOOH nanosheets were dispersed in the water. The concentration of AlOOH nanosheets dispersion liquid was 1 mg mL^−1^. The AlOOH films were prepared by drop casting of the AlOOH dispersion liquid onto a ITO substrate. After drop casting, the sample was dried at room temperature, and this process was repeated for three times. Then, about 300 nm AlOOH film was obtained. The Pt electrode is grown by magnetron sputtering with the assistance of a shadow mask with a diameter of 10 µm. In particular, the ITO:PDMS substrate is customized by Le Lin Technology Development Co., Ltd.

### Cell culture

Raw 264.7 cells (5 × 10^3^ cells per well, 100 µL per well) were cultured in Dulbecco’s modified Eagle’s medium (DMEM; Gibco) containing 10% fetal bovine serum (Gibco) and 1% penicillin/streptomycin (Gibco). Raw264.7 cells were incubated at a humidity atmosphere containing 5% CO_2_ at 37 °C for 24, 48, and 72 h. For AlOOH nanosheets sterilized by 120 ℃, aqueous extractions with different concentrations (50, 100, 150 and 200 μg mL^−1^) were synthesized for raw 264.7 cells cell culture. Cell viability is equal to the absorbance ratio of the experimental group and the control group.

### Cellular viability test (CCK-8) assay

At indicated time points, the samples were washed three times with PBS. Then 360 µL of medium with 40 µL of CCK-8 solution was added to each disk. After incubation for 2 h at 37 ℃, 100 µL of the incubated solution was transformed to a new 96-well plate, and the optical density (OD) value of the solution was measured with an ELISA plate reader (Varioskan Flash 3001, Thermo, Finland) at 450 nm wavelength.

### Animal experiments

For biocompatibility studies, five 6-week-old male C57BL/6 mice were used. Dorsal hair was removed with the help of a 0.1 mm animal hair clipper. All animal studies were performed under protocols approved by the Shanghai Jiao Tong University Institutional Animal Care and Use Committee (Approval No. B-2021-009). The skin was cleaned with alcohol wetted cotton swab for 3 times. 4% Chloral Hydrate was used to induce anesthesia. For subcutaneous implantation, a small incision was made in the posterior neck and Pt/AlOOH/ITO was inserted into the wound. After that, the incision was closed with absorbable sutures. After implanting for 15, 30, 45 and 60 days, respectively, mice were sacrificed and the skin close to Pt/AlOOH/ITO were harvested and fixed with paraformaldehyde (4% in PBS) for histological analyses. To evaluate if the Pt/AlOOH/ITO is harmful to the major organs, spleen, liver, lung, rein, and heart samples after 60 days implanting were harvested and fixed with paraformaldehyde (4% in PBS) for histological analyses.

### Electrical measurements

All electrical characterizations of the device were performed at ambient condition using a probe station and a Keithley 4200-SCS semiconductor parameter analyzer system equipped with our programming test software. The voltage signals designed for specific learning rules are applied to the ITO electrode, and the Pt electrode is grounded.

### Material characterizations

The crystallization and phase structure of the fabricated AlOOH was carried out by X-ray diffraction (XRD) system (Empyren, PANalytical) and surface chemical states were examined by X-ray photoelectron spectroscopy (XPS, 250Xi ESCALAB). Fourier transform infrared (FTIR) spectra were obtained using a Thermo Fisher Scientific ls-50 spectrometer in the range 400–4000 cm^−1^ at room temperature. The morphologies of the AlOOH were observed by scanning electron microscopy (SEM, TESCAN MIRA3 LMU). The high-resolution transmission electron microscope (HRTEM) was measured using a FEI Tecnai G2 F20. All the electrical measurements were performed on a probe station connected to a Keithley 4200-SCS semiconductor characterization system.

### DFT method

The structural optimizations and electronic structure calculations are performed based on DFT as implemented in the Vienna Ab Initio Simulation Package (VASP) code [55], based on the projector augmented wave (PAW) method with a cutoff energy of 600 eV [56]. The configuration of AlOOH was fully optimized [55]. The generalized gradient form (GGA) of the exchange–correlation functional (Perdew-Burke-Ernzerhof 96, PBE) was adopted [57, 58]. A revised PBE generalized gradient approximation was used for the exchange–correlation [59, 60]. PBE sol functional has been introduced to improve the equilibrium properties of solids [61]. Valence-core interactions were described by projector-augmented-wave (PAW) pseudopotentials [62]. The Brillouin zone sampling is carried out using the (3 × 3 × 1) Monkhorst–Pack grids for surface and Gamma for the structure [56]. The convergence tolerance of energy is 1 × 10^–5^ eV, maximum force is 0.002 eV·Å^−1^, and maximum displacement is 0.002 Å [56].

### Supplementary Information

**Additional file 1: Figure S1.** The performance of various biomemristor devices were summarized. **Figure S2.** Presenting Different I-V Curves of under Different Compliance Currents (ICC). **Table S1.** Comparison of the memristive performance of implantable memristors. **Figure S3.** Morphology Characterization of AlOOH Film and AlOOH Nanosheets. **Figure S4.** Al_2_O_3_ Nanosheets Memristors. **Figure S6.** Corresponding conduction energy band profiles for the set (left) and reset (right). Formula Unit Conversion of formula/Eq. (2). **Table S2.** Carrier Mobility of Metal Oxides. **Table S3.** Raw data for the Cell Counting Kit-8 (CCK-8) tests. **Figure S7.** The Comparison Between AlOOH and Other Representative Biomemristors.

## Data Availability

All data in this study are available from the corresponding author upon reasonable request.
