# Supplementary material for: Flexible multilevel nonvolatile biocompatible memristor with high durability
Source: J Nanobiotechnology. 2023 Oct 13;21:375. doi: 10.1186/s12951-023-02117-5 (PMC10576337; doi:10.1186/s12951-023-02117-5)
Supplement: Supplementary file 1 — Additional file 1: Figure S1. The performance of various biomemristor devices were summarized. Figure S2. Presenting Different I-V Curves of under Different Compliance Currents (ICC). Table S1. Comparison of the memristive performance of implantable memristors. Figure S3. Morphology Characterization of AlOOH Film and AlOOH Nanosheets. Figure S4. Al2O3 Nanosheets Memristors. Figure S6. Corresponding conduction energy band profiles for the set (left) and reset (right). Formula Unit Conversion of formula/Eq. (2). Table S2. Carrier Mobility of Metal Oxides. Table S3. Raw data for the Cell Counting Kit-8 (CCK-8) tests. Figure S7. The Comparison Between AlOOH and Other Representative Biomemristors. [file 12951_2023_2117_MOESM1_ESM.docx]

Additional file

**Flexible Multilevel Nonvolatile Biocompatible Memristor with High Durability**

Xiaoping Chen, Xu Zhao, Xiaozhong Huang, Xiu-Zhi Tang, Ziqi Sun*, Da-Long Ni*, Hailong Hu^*^_,_ Jianling Yue^*^

X. Chen, X. Zhao, Prof. J. Yue, Prof. X. Huang

Powder Metallurgy Research Institute, Central South University, Changsha 410083, China

Email: [jlyue2010@csu.edu.cn](mailto:jlyue2010@csu.deu.cn)

Prof. Z. Sun

School of Chemistry and Physics, QUT Centre for Materials Science, Queensland University of Technology, Brisbane, QLD 4001, Australia

Email: ziqi.sun@qut.edu.au

Prof. D.-L. Ni

Department of Orthopaedics, Shanghai Key Laboratory for Prevention and Treatment of Bone and Joint Diseases, Shanghai Institute of Traumatology and Orthopaedics, Ruijin Hospital, Shanghai Jiao Tong University School of Medicine, Shanghai 200025, China

Email: ndl12353@rjh.com.cn

Associate Prof. X.-Z. Tang

Research Institute of Aerospace Technology, Central South University, Changsha 410083, China

Associate Prof. H. Hu

State Key Laboratory of High Performance Ceramics and Superfine Microstructure, Research Institute of Aerospace Technology, Central South University, Changsha 410083, China

Email: hailonghu@csu.edu.cn

Contents

Note 1. the Performance of Most Biomemristors


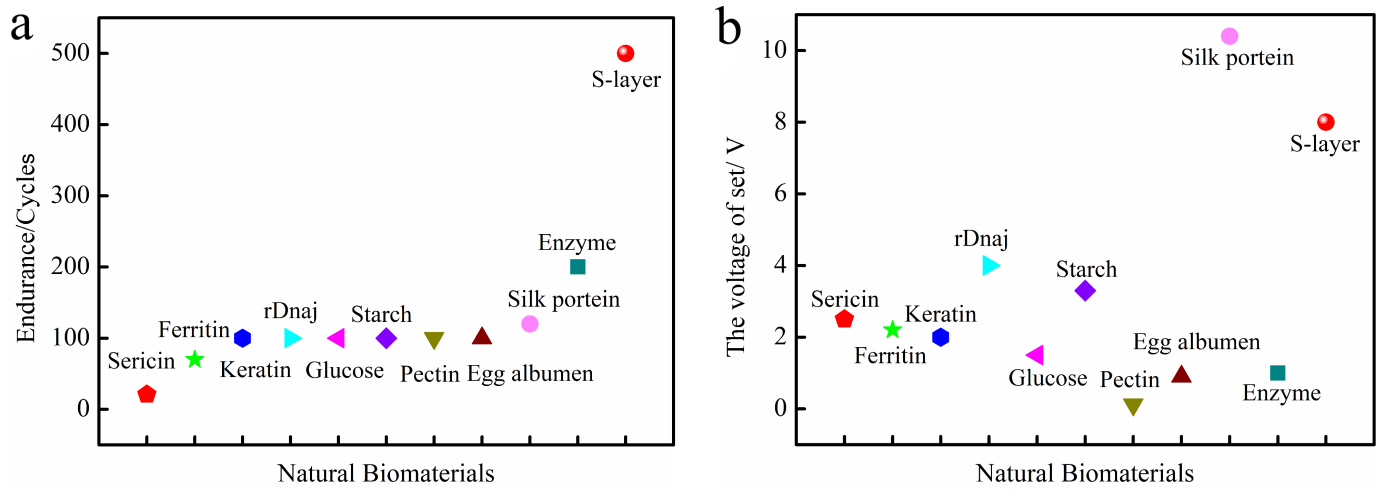
**Figure S1.** The performance of various biomemristor devices were summarized.^[1-11]^ a) endurance. b) the voltage of set.

Note 2. Presenting Different I-V Curves of under Different Compliance Currents (I_CC_)


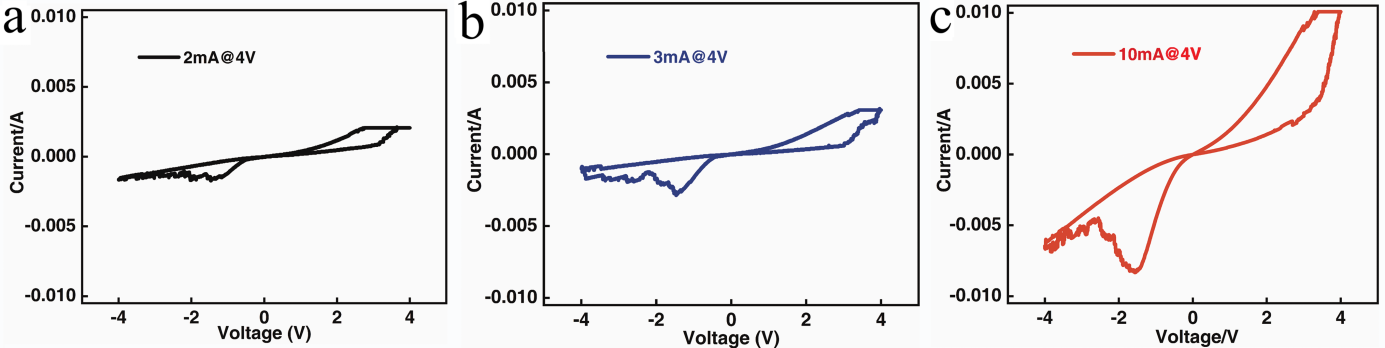


**Figure S2.** Figure shows the triangular wave periodic voltage along 0 V→4 V→0 V→-4 V→0 V, and three different I-V curves measured under the conditions of I_CC_ of 2 mA(a), 3 mA(b), and 10 mA(c).

Note 3. Comparison of the Memristive Performance of Biomemristors

**Table S1.** Comparison of the memristive performance of implantable memristors

| Device structure | On/off ratio | V_Set_/V_Reset_  [V] | The range of I_CC_[mA] | Retention  [s] | Endurance  [Cycles] | Multilevel | Ref. |
| --- | --- | --- | --- | --- | --- | --- | --- |
| Ag/ Enzyme /Pt | 10^6^ | 1/–1.3 | / | 10^4^ | 200 | No | ^[1]^ |
| Cu/rDnaj/Pt | 10^6^ | 0.12/–0.08 | 10^-3^,10^-2^  (22 cycles) | 10^6^ | 100 | Yes | ^[2]^ |
| Ag/ Keratin /FTO | 10^3^ | 1.5/–1.5 | 1-5 | 10^4^ | 100 | Yes | ^[3]^ |
| Al/S-layer/ITO | 6.2 | 8/–8 | / | 10^3^ | 500 | No | ^[4]^ |
| Au/Starch/ITO | 10^3^ | 0.9/-1.3 | 0.1 | 10^4^ | / | No | ^[5]^ |
| Ag/Pectin/FTO | 10^2^ | 3.3/-4.5 | / | 10^3^ | 100 | No | ^[6]^ |
| Pt/Ferritin/Pt | 10^5^ | 2.2/–1.1 | 5×10^-3^，10^-4^，10^-5^ | 10^3^ | 70 | Yes | ^[7]^ |
| Ag/Sericin/Au | 10^6^ | 2.5/–0.8 | 0.1,1,10  (21 cycles) | 10^3^ | 21 | Yes | ^[8]^ |
| W/egg albumen /ITO | 10^2^ | 2/7 | / | 10^3^ | 100 | No | ^[9]^ |
| Al/Glucose/ITO | 10^3^ | 4/-4 | 1 | 10^4^ | 100 | No | ^[10]^ |
| Al/Silk Fibroin  /ITO | 10 | 10.4/–11.5 | / | 10^3^ | 120 | No | ^[11]^ |
| Ag/HfO_2_/BST:Au/Pt | 10^3^ | 0.18/-0.2 | 0.1-0.2 | 10^4^ | 50 | Yes | ^[12]^ |
| Al/CDs- Silk Fibroin/ITO | - | 3/–1 | 1,10,100 | 10^6^ | 100 | Yes | ^[13]^ |
| Ag/Ag-Silk Fibroin /ITO | 10^3^ | 0.3/–0.18 | / | 10^4^ | 100 | No | ^14]^ |
| W/MgO/ZnO/Mo | 8 | 1.32/-1.32 | / | 10^4^ | 300 | No | ^[15]^ |
| Ag/ Silk Fibroin /Au | 10^5^ | 1.7/–1.2 | / | 10^4^ | 30 | No | ^[16]^ |
| Ag/Au-Silk Fibroin /ITO | 10^2^ | 0.4/–0.2 | / | 10^4^ | 100 | Yes | ^[17]^ |
| Ag/egg albumen/ITO | 10^2^ | 0.6/-0.7 | 3 | 10^4^ | 50 | No | ^[18]^ |
| Au/Silk Fibroin /Pt | 10^4^ | 3/–1.7 | 10^-3^ | 10^3^ | 30 | No | ^[19]^ |
| Pt/AlOOH/ITO | 10^2(^max^)^ | 2/-1.2 | 1,2,4,6  (1400 cycles) | 10^3^ | 10^3^ | Yes | This work |

Note 4. Morphology Characterization of AlOOH Film and AlOOH Nanosheets


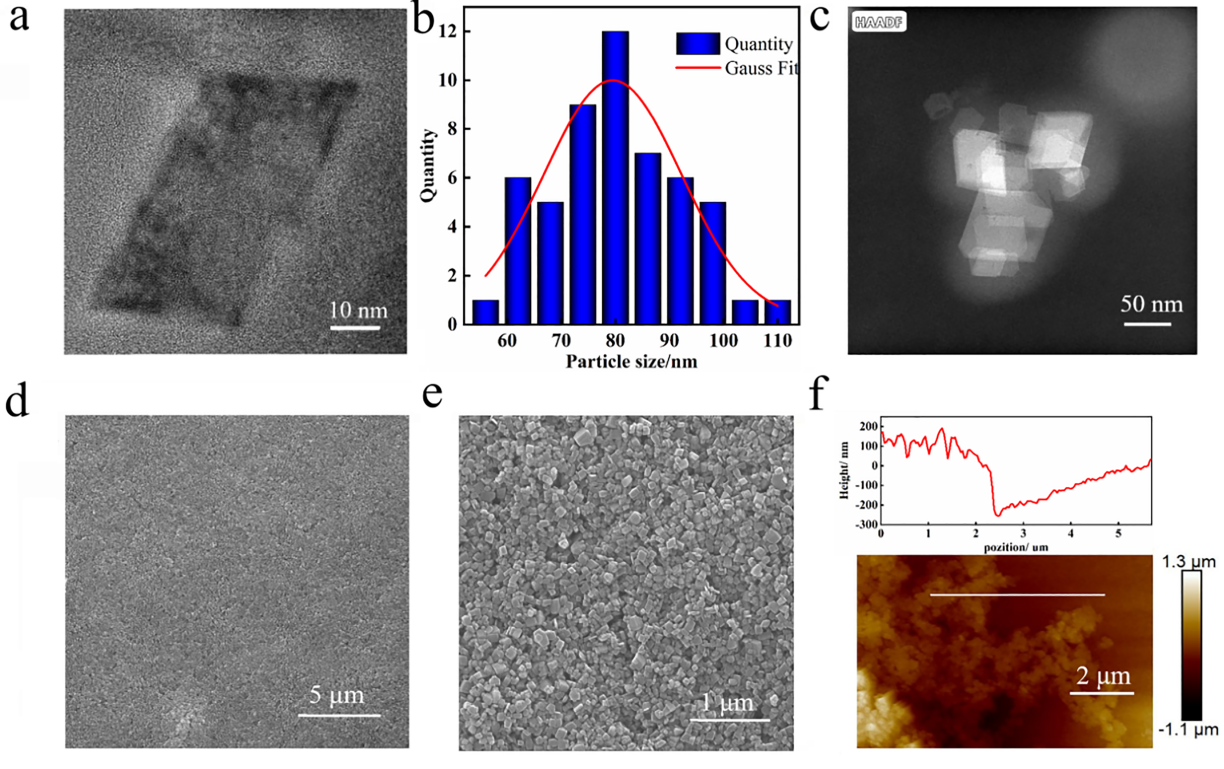


**Figure S3.** Morphology characterization of AlOOH film and AlOOH nanosheets. a) Low-magnification TEM image of one AlOOH nanosheet. b) Statistics on the size of AlOOH nanosheets in Figure 2a. c) The image of HAADF-STEM, showing that AlOOH nanosheets is a layered structure. d-e) SEM topography image of AlOOH film at different magnifications. f) AFM confirming the high quality of film is preserved after the device fabrication. Inset is thickness of AlOOH film edge area.

Note 5. Al_2_O_3_ Nanosheets Memristors


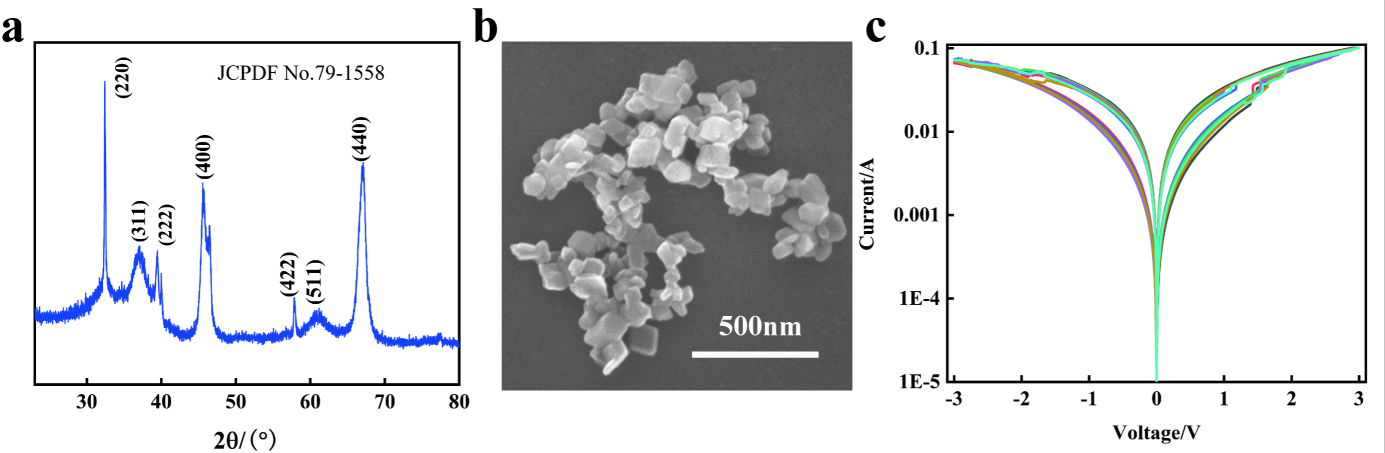


**Figure S5.** a) XRD pattern of Al_2_O_3_ nanosheets obtained after calcination. b) Al_2_O_3_ nanosheets inherited the AlOOH morphology. c) the I-V curves of Pt/Al_2_O_3_ nanosheets/ITO device.

Note 6. Corresponding Conduction Energy Band


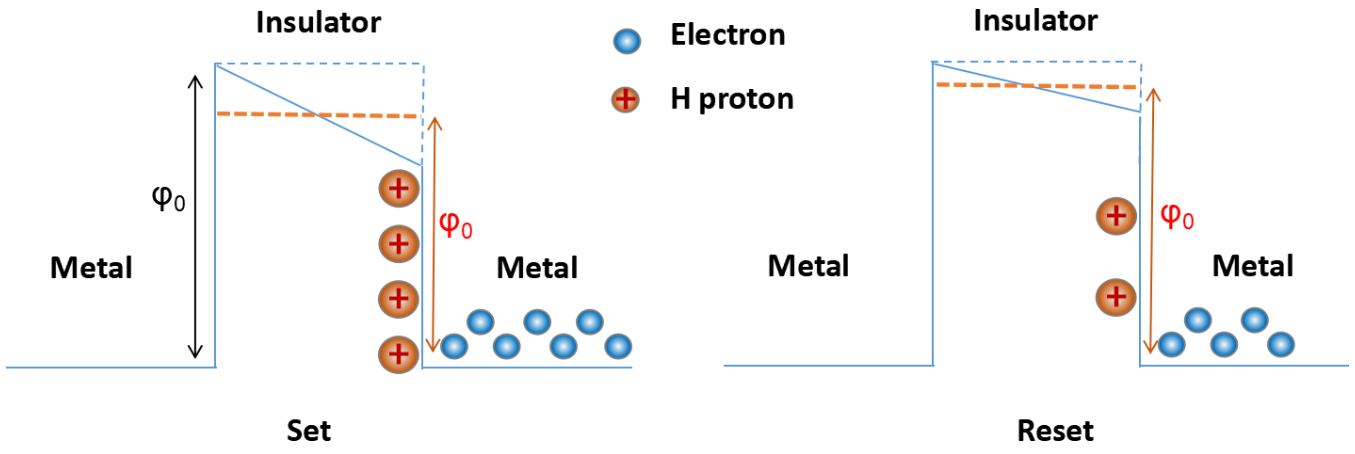
Figure S6. Corresponding conduction energy band profiles for the set (left) and reset (right) .

Note 7. Formula Unit Conversion of formula/equation (2)

$\frac{C\cdot J^{3}\cdot s^{3}\cdot\frac{J}{m^{3}}}{\frac{J}{K}\cdot K\cdot\mathrm{kg}\cdot\mathrm{kg}\cdot J^{2}}$=$\frac{C\cdot J^{3}\cdot s^{3}}{m^{2}\cdot\mathrm{kg}^{2}}$ =$\frac{C\cdot J}{\frac{m^{2}\cdot\mathrm{kg}^{2}}{s^{3}} \cdot\frac{s}{s} \cdot\frac{m^{2}}{m^{2}}}$

= $\frac{C\cdot J}{\frac{s}{m^{2}} \cdot\frac{m^{4}\cdot\mathrm{kg}^{2}}{s^{2}}}$ = $\frac{m^{2}}{s}\cdot\frac{C}{J}$

= $m^{2}\cdot s^{-1}{\cdot V}^{-1}$

Where 1 V = 1 J$\cdot$C^-1^, 1 J = kg$\cdot$m^2^$\cdot$s^-2^

Note 8. Carrier Mobility of Metal Oxides

**Table S2.** Carrier Mobility of Metal Oxides

| Materials | Carrier mobility (cm^2^ V ^−1^ s ^−1^ ) |
| --- | --- |
| Ti_0.91_O_2_^[20]^ | 500 |
| ZnO thin film^[21]^ | 400 |
| In_2_O_3_ nanowire^[22]^ | 70-250 |
| MoS_2_/Al_2_O_3_^[23]^ | 41.2 |
| This work | 240 |

Note 9. the Cell Counting Kit-8 (CCK-8) Tests

**Table S3.** Raw data for biocompatibility experiments

| Concentration  [μg·mL^−1^)] | The OD value of 1 days | Standard deviation | The OD value of 2 days | Standard deviation | The OD value of 3 days | Standard deviation | The OD value of 5 days | Standard deviation |
| --- | --- | --- | --- | --- | --- | --- | --- | --- |
| 0 | 0.571375 | 0.032771727 | 1.435375 | 0.089156464 | 1.6935 | 0.132009259 | 1.691625 | 0.080104012 |
| 50 | 0.745125 | 0.025242986 | 1.41025 | 0.171741893 | 1.660625 | 0.069918901 | 1.555125 | 0.07115154 |
| 100 | 0.85775 | 0.089406376 | 1.39825 | 0.092503153 | 1.48625 | 0.187792882 | 1.428125 | 0.179780383 |
| 150 | 0.854625 | 0.109526189 | 1.6225 | 0.30531441 | 1.382875 | 0.122755799 | 1.48575 | 0.121078258 |
| 200 | 0.8945 | 0.104169733 | 1.4865 | 0.106485262 | 1.50975 | 0.124795611 | 1.593125 | 0.084364866 |

Note 10. The Comparison Between AlOOH and Other Representative Biomemristors


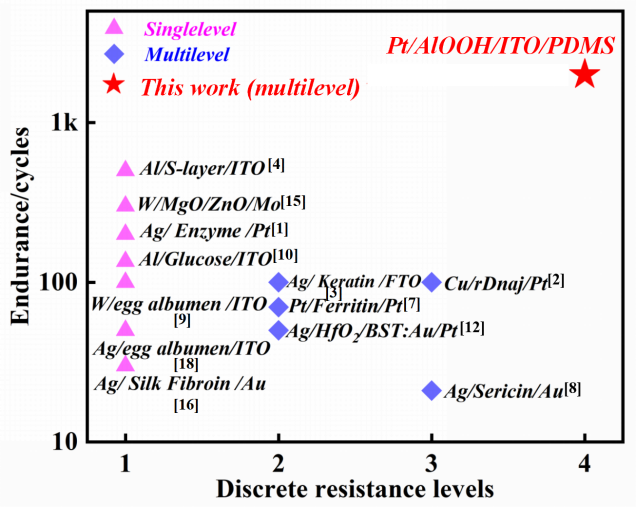


**Figure S7.**The comparison of the endurance and discrete resistance levels between AlOOH and other representative biomemristors.^[1,3-4,7-10,12,15-16,18]^

References

[1] H. Baek, C. Lee, K. I. Lim, J. Cho, Resistive switching memory properties of layer-by-layer assembled enzyme multilayers. *Nanotechnol.* **2012**, *23*, 155604.

[2] S. K. Jang, S. Kim, M. S. Salman, J. R. Jang, Y. M. Um, L. Tan, J. H. Park, W. S. Choe, S. Lee, Harnessing recombinant DnaJ protein as reversible metal chelator for a high-performance resistive switching device. *Chem. Mater.* **2018**, *30*, 781.

[3] Q. Lin, S. Hao, W. Hu, M. Wang, Z. Zang, L. Zhu, J. Du, X. Tang, Human hair keratin for physically transient resistive switching memory devices. *J. Mater. Chem. C* **2019**, *7*, 3315.

[4] A. Moudgil, N. Kalyani, G. Sinsinbar, S. Das, P. Mishra, S-layer protein for resistive switching and flexible nonvolatile memory device. *ACS Appl. Mater. Interfaces* **2018**, *10*, 4866.

[5] N. R. Hosseini, J. S. Lee, Controlling the resistive switching behavior in starch-based flexible biomemristors. *ACS Appl. Mater. Interfaces* **2016**, *8*, 7326.

[6] B. Sun, X. Zhang, G. Zhou, P. Li, Y. Zhang, H. Wang, Y. Xia, Y. Zhao, An organic nonvolatile resistive switching memory device fabricated with natural pectin from fruit peel. *Org. Electron.* **2017**, *42*, 181.

[7] C. Zhang, J. Shang, W. Xue, H. Tan, L. Pan, X. Yang, S. Guo, J. Hao, G. Liu, R.-W. Li, Convertible resistive switching characteristics between memory switching and threshold switching in a single ferritin-based memristor. *Chem. Commun.* **2016**, *52*, 4828.

[8] H. Wang, F. Meng, Y. Cai, L. Zheng, Y. Li, Y. Liu, Y. Jiang, X. Wang, X. Chen, Sericin for resistance switching device with multilevel nonvolatile memory. *Adv. Mater.* **2013**, *25*, 5498.

[9] X. Yan, X. Li, Z. Zhou, J. Zhao, H. Wang, J. Wang, L. Zhang, D. Ren, X. Zhang, J. Chen, C. Lu, P. Zhou, Q. Liu, Flexible transparent organic artificial synapse based on the tungsten/egg albumen/indium tin oxide/polyethylene terephthalate memristor. *ACS Appl. Mater. Interfaces* **2019**, *11*, 18654.

[10] S. P. Park, Y. J. Tak, H. J. Kim, J. H. Lee, H. Yoo, H. J. Kim, Analysis of the bipolar resistive switching behavior of a biocompatible glucose film for resistive random access memory. *Adv. Mater.* **2018**, *30*, 1800722.

[11] M. K. Hota, M. K. Bera, B. Kundu, S. C. Kundu, C. K.Maiti, A natural silk fibroin protein‐based transparent bio‐memristor. *Adv. Funct. Mater.*  **2012**, *22*, 4493.

[12] X. Li, L. Zhang, R. Guo, J. Chen, X. Yan, A Flexible Transient Biomemristor Based on Hybrid Structure HfO_2_/BSA:Au Double Layers. *Adv. Mater. Technol.* **2020**, *5*, 2000191.

[13] Z. Lv, Y. Wang, Z. Chen, L. Sun, J. Wang, M. Chen, Z. Xu, Q. Liao, L. Zhou, X. Chen, Phototunable biomemory based on light‐mediated charge trap. *Adv. Sci.* **2018**, *5*, 1800714.

[14] C. Shi, J. Wang, M. L. Sushko, W. Qiu, X. Yan, X. Y. Liu, Silk Flexible Electronics: From Bombyx mori Silk Ag Nanoclusters Hybrid Materials to Mesoscopic Memristors and Synaptic Emulators. *Adv. Funct. Mater.* **2019**, *29*,1904777.

[15] B. Dang, Q. Wu, F. Song, J. Sun, M. Yang, X. Ma, H. Wang , Y. Hao, A bio-inspired physically transient/biodegradable synapse for security neuromorphic computing based on memristors. *Nanoscale* **2018**, *10*, 20089.

[16] H. Wang, B. Zhu, H. Wang, X. Ma, Y. Hao, X. Chen, Ultra‐Lightweight Resistive Switching Memory Devices Based on Silk Fibroin. *Small* **2016**, *12*, 3360.

[17] Y. Xing, C. Shi, J. Zhao, W. Qiu, N. Lin, J. Wang, X. B. Yan, W. D. Yu, X. Y. Liu, Mesoscopic‐Functionalization of Silk Fibroin with Gold Nanoclusters Mediated by Keratin and Bioinspired Silk Synapse. *Small* **2017**, *13*,1702390.

[18] J. X. Zhu, W. L. Zhou, Z. Q. Wang, H. Y. Xu, Y. Lin, W. Z. Liu, J. G. Ma, Y. C. Liu, Flexible, transferable and conformal egg albumen based resistive switching memory devices. *RSC Adv.* **2017**, *7*, 32114.

[19] J. Yong, B. Hassan, Y. Liang, K. Ganesan, R. Rajasekharan, R. Evans, G. Egan, O. Kavehei, J. Li, G. Chana, B. Nasr, E. Skafidas, Hydrogen enhances strength and ductility of an equiatomic high-entropy alloy. *Sci. Rep.* **2017**, *7*,1.

[20] M. Osada, T. Sasaki, Two‐dimensional dielectric nanosheets: novel nanoelectronics from nanocrystal building blocks. *Adv. Mater.* **2012**, *24*, 210.

[21] I. Gonzalez-Valls, M. Lira-Cantu, Vertically-aligned nanostructures of ZnO for excitonic solar cells: a review. *Energy Environ. Sci.* **2009**, *2*, 19.

[22] H. Huang, B. Liang, Z. Liu, X. Wang, D. Chen, G. Shen, Metal oxide nanowire transistors. *J. Mater. Chem.* **2012**, *22*, 13428.

[23] S. Y. Kim, S. Park, W. Choi, Enhanced carrier mobility of multilayer MoS2 thin-film transistors by Al2O3 encapsulation. *Appl. Phys. Lett*. **2016**, *109*, 152101.
